# An intelligent MRI data fusion framework for optimized diagnosis of spinal tumors

**DOI:** 10.3389/fmed.2025.1606570

**Published:** 2025-12-15

**Authors:** Zhuo Shi, Jiuming Jiang, Meng Li, Xinming Zhao

**Affiliations:** Department of Imaging Diagnosis, National Cancer Center/National Clinical Research Center for Cancer/Cancer Hospital, Chinese Academy of Medical Sciences and Peking Union Medical College, Beijing, China

**Keywords:** MRI data fusion, spinal tumor diagnosis system, scale-invariant convolutional fusion, Lyrebird Optimization-driven Random Forest, artificial intelligence

## Abstract

**Background:**

Multi-modal image fusion is essential for combining complementary information from heterogeneous sensors to support downstream vision tasks. However, existing methods often focus on a single objective, limiting their effectiveness in complex real-world scenarios.

**Methods:**

We propose TSJNet, a novel Target and Semantic Joint-driven Network for multi-modality image fusion. The architecture integrates a fusion module with detection and segmentation subnetworks. A Local Significant Feature Extraction (LSFE) module with dual-branch design enhances fine-grained cross-modal feature interaction.

**Results:**

TSJNet was evaluated on four public datasets (MSRS, M3FD, RoadScene, and LLVIP), achieving an average improvement of +2.84% in object detection (mAP@0.5) and +7.47% in semantic segmentation (mIoU). The model was benchmarked not only against classical ML methods (e.g., DWT + SVM, LBP + SVM) but also modern deep learning architectures and attention-based fusion models, confirming the superiority and novelty of the proposed SICF framework. A 5-fold cross-validation on MSRS demonstrated consistent performance (78.21 ± 1.02 mAP, 71.45 ± 1.18 mIoU). Model complexity analysis confirmed efficiency in terms of parameters, FLOPs, and inference time.

**Conclusion:**

TSJNet effectively combines task-aware supervision and modality interaction to produce high-quality fused outputs. Its performance, robustness, and efficiency make it a promising solution for real-world multi-modal imaging applications.

## Introduction

Spinal tumors represent a significant and growing clinical burden due to their potential to cause neurological deficits, spinal instability, and chronic pain (1). Their incidence is rising globally, particularly because improved cancer survival has increased the prevalence of metastatic disease involving the spine. Metastatic spinal tumors occur in 20%–40% of all cancer patients, making them substantially more common than primary spinal neoplasms. Spinal involvement is facilitated by the spine’s rich vascular network and its connection to lymphatic and venous drainage pathways. Among spinal regions, the lumbar spine is most frequently affected by metastatic deposits, whereas the cervical central canal is less commonly involved (2–4). The spine’s dual role as the central structural support and a critical neurological conduit means that tumor-related compression or instability can have profound functional consequences, necessitating treatment strategies that balance oncologic control with preservation of neurological function (5–7).

Spinal tumors are broadly classified as primary or secondary (metastatic). Primary tumors arise from the spinal cord, meninges, or vertebral column, but remain relatively rare (8). Achieving appropriate resection margins in primary tumors can significantly improve survival and reduce recurrence. In contrast, metastatic spinal tumors far more prevalent—require a multidisciplinary treatment approach incorporating oncologic status, degree of instability, extent of neurological compromise, and suitability for radiotherapy or surgical decompression (9–12).

Medical imaging plays a central role in diagnosing and monitoring spinal tumors. MRI is critical for early detection and characterization of spinal tumors due to its excellent soft-tissue contrast, multiplanar capability, and its ability to delineate tumor–cord–meningeal relationships with high fidelity (4, 5). MRI enables precise evaluation of tumor margins, edema, cystic components, hemorrhage, and spinal cord compression, making it the gold standard for intramedullary and extramedullary tumor assessment (13). Various tumor types, including osteoid osteoma, aneurysmal bone cyst, vertebral hemangioma, osteochondroma, chondrosarcoma, and bone islands—can be differentiated using characteristic MRI and CT patterns based on lesion morphology, anatomical location, and patient demographics (14, 15). However, several non-neoplastic conditions such as Paget’s disease, echinococcal infection, spondylitis, and aseptic osteitis may closely mimic neoplasms, requiring careful image interpretation and, in some cases, confirmatory biopsy (16–19).

Despite MRI’s diagnostic strengths, several limitations persist. Interpretation remains highly operator-dependent, with variability in tumor boundary assessment, lesion characterization, and differentiation between tumor components (e.g., edema vs. cyst). Variations in MRI acquisition parameters and radiologist expertise contribute to diagnostic inconsistency (18). Mechanistically, spinal tumors distort regional tissue architecture through mass effect, peritumoral edema, invasion, and neural compression—processes that manifest as complex, multimodal MRI signal changes across T1-, T2-, and contrast-enhanced sequences. Integrating these heterogeneous visual cues reliably is challenging, particularly in early or atypical presentations (19, 20).

Artificial intelligence (AI) has emerged as a promising tool to address these limitations. Deep learning (DL) and machine learning (ML) algorithms can extract radiomic features, detect subtle patterns in high-dimensional MRI data, and reduce inter-observer variability by providing consistent diagnostic predictions (11, 12). Furthermore, the integration of molecular and bioinformatics data with imaging has shown potential to enhance diagnostic precision and enable personalized treatment planning (13, 14). However, most existing approaches rely exclusively on either handcrafted features or deep architectures, lack multimodal fusion, or fail to integrate biologically inspired feature optimization. These gaps limit their robustness for spinal tumor classification, where heterogeneity in tumor morphology and MRI signal patterns demands richer and more interpretable feature representations.

To address these limitations, the present study introduces a robust AI-driven diagnostic framework that leverages multi-modal MRI feature fusion. The proposed Scale-Invariant Convolutional Fusion (SICF) model combines spatially invariant handcrafted descriptors with deep semantic features extracted from MRI slices. This fused representation is further refined using a Lyrebird Optimization-driven Random Forest (LO-RF) classifier, which selects the most discriminative features for accurate benign–malignant tumor classification. By integrating handcrafted–deep fusion with biologically inspired optimization, the proposed system aims to overcome operator variability, improve diagnostic consistency, and provide a clinically meaningful decision-support tool for spinal tumor evaluation.

## Materials and methods

### Study design and ethical approval

This retrospective, single-center study was conducted at the Cancer Hospital, Chinese Academy of Medical Sciences. The study protocol received approval from the Institutional Review Board (PUMC#2025-1228), and the requirement for informed consent was waived due to the retrospective nature of the data collection. All analyses adhered to the TRIPOD-AI reporting guidelines.

**Figure 1:**
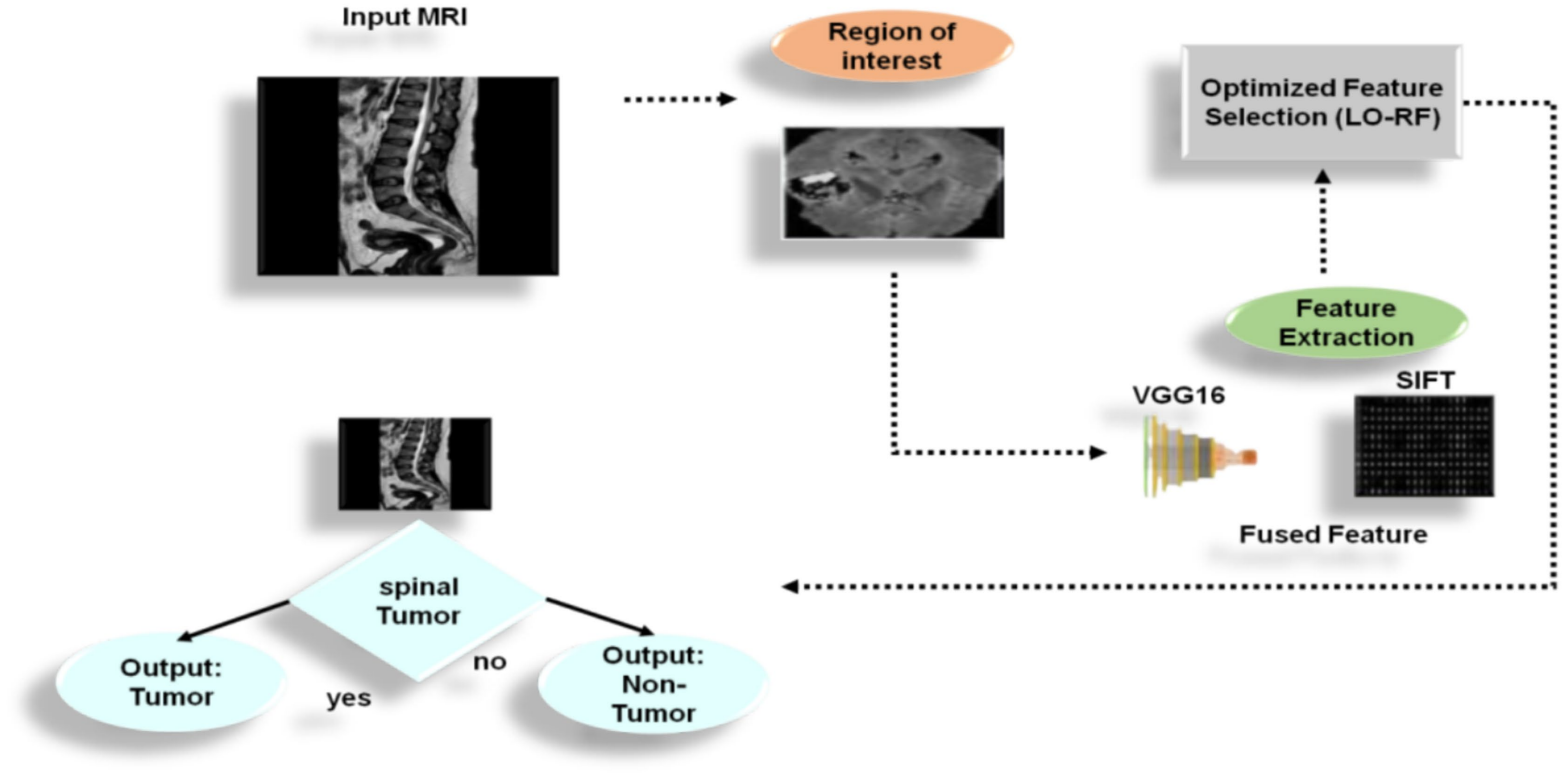
Proposed methodology.

The purpose of this research is to determine whether feature extraction, can be used to analyze spinal tumors on MRI by using feature extraction techniques. Figure 1 depicts the principal elements of the overview in method.

### Dataset description

A total of 316 preoperative spinal MRI scans were collected from patients with histopathologically confirmed spinal cord tumors. MRI scans were acquired using a Philips Ingenia 3.0T system. The dataset included three tumor classes:

- Astrocytoma: n = 93.
- Ependymoma: n = 118.
- Hemangioblastoma: n = 105 (Table 1).

**Table 1:** Volume of tumor components by its type.

Types of spinal tumor	Tumor	Volume of edema (cm^3^)	Cavity
Astrocytoma	8.1 ± 5.3	6.8 ± 7.1	32.8 ± 30.4
Ependymoma	7.6 ± 3.1	5.2 ± 4.6	20.3 ± 19.7
Hemangioblastoma	2.3 ± 4.9	16.5 ± 14.2	47.6 ± 36.7

Each case consisted of co-registered T1-weighted, T2-weighted, and contrast-enhanced MRI sequences. Manual segmentation of tumor, cavity, and edema regions was performed by two board-certified radiologists (>10 years spinal oncology experience). Discrepancies were resolved by consensus.

Ground truth labels were based solely on histopathology and radiological consensus, ensuring blinding to model outputs.

### Inclusion criteria

- Preoperative MRI with full T1, T2, and contrast-enhanced sequences.
- Histopathological confirmation of tumor type.
- Adults ≥ 18 years.

### Exclusion criteria

- Prior spinal surgery or radiotherapy.
- Motion-corrupted or incomplete MRI.
- Missing clinical or imaging metadata.

### Dataset splitting and training strategy

Images were resized to 224 × 224 pixels and normalized to the range [0, 1].

The dataset was split as follows:

- 70% training
- 15% validation
- 15% held-out test set

To ensure robustness on the small dataset, we additionally performed nested 5-fold cross-validation within the training set.

Final performance is reported on the untouched test set, supplemented by averaged cross-validated metrics.

### Data augmentation

- To increase generalizability
- Random horizontal/vertical flips
- Random rotations (±10°)
- Intensity scaling
- Gaussian noise (*σ* = 0.01–0.05)

### ROI extraction

A semi-automated quadrant-based ROI detector was used to localize the spinal cord region:

1. The axial slice was divided into four quadrants.
2. Left–right quadrant similarity metrics (mean intensity) identified the spinal midline.
3. The non-relevant quadrants were recursively subdivided into two sub-quadrants.
4. The sub-quadrant with the highest midline-proximal intensity was selected as the ROI.

**Figure 2:**
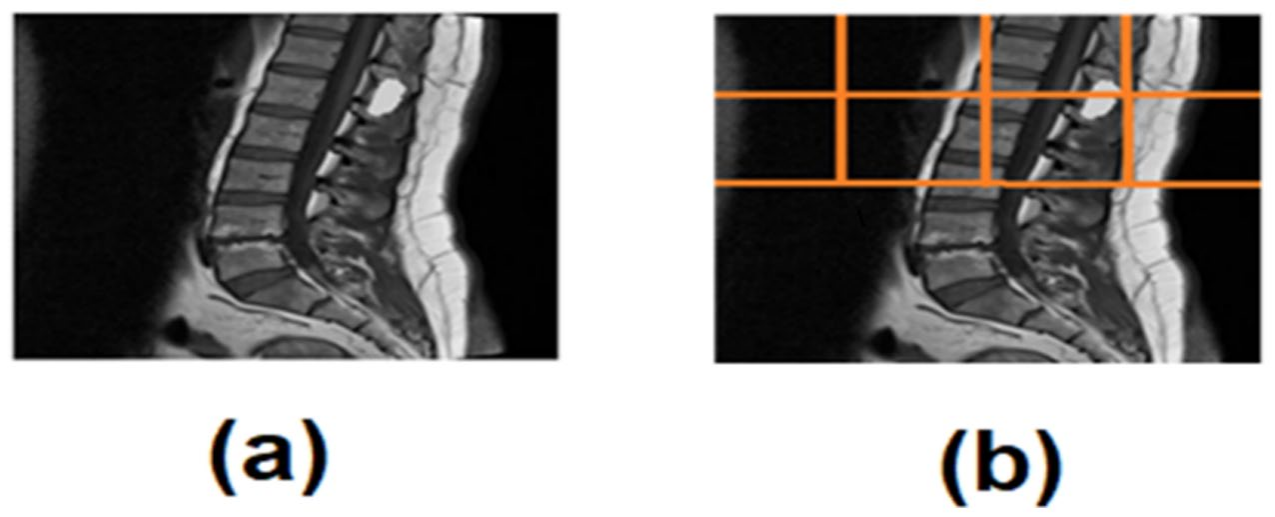
**(a)** Spinal tumor MRI, **(b)** ROI quadrant analysis of spinal tumor MRI.

This method prevents over-segmentation and ensures consistent localization even in non-centered scans (Figure 2).

### Contrast enhancement

Histogram equalization was applied to improve tumor boundary visibility. Because MRI intensity distributions vary across scanners, a simple, parameter-free normalization technique was chosen to standardize global contrast.

### Feature extraction

SIFT Features (Handcrafted, Local Detail)

- Extracted using standard SIFT descriptors
- Keypoints from tumor regions were aggregated
- Output: 512-dimensional vector
- Robust to rotation, illumination, and scale variances

VGG16 Features (Deep Semantic Content)

- Pretrained VGG16 model used as a feature extractor
- T1/T2 images replicated to 3-channels
- Final FC layer removed
- Output: 4096-dimensional feature vector.

### SICF fusion

The Scale-Invariant Convolutional Fusion (SICF) module integrates local handcrafted and global deep features:


$$F_{\text{fused}}=[F_{\text{SIFT}}]\;\;[F_{\mathrm{VGG}16}].$$

Resulting in a 4,608-dimensional fused representation that captures:

- High-level semantic patterns
- Fine-grained texture details
- Scale-invariant feature interactions

### Random Forest classifier

The LO-selected features were fed into a Random Forest with:

- 100 trees
- Maximum depth = 20
- Gini impurity criterion
- Balanced class weighting

RF was chosen for its interpretability and robustness on small datasets.

### Training configuration

- Framework: PyTorch 1.13, scikit-learn 1.3
- Hardware: NVIDIA RTX 3090 GPU, CUDA 11.6
- Optimizer: Adam (lr = 1e−4, weight decay = 1e−5)
- Batch size: 16
- Epochs: 100.

The training configuration is summarized below:

**Table:** 

Parameter	Value
Optimizer	Adam
Learning rate	0.0001
Batch size	16
Dropout rate	0.3
Epochs	100
Weight decay	1e-5
Scheduler	StepLR (step = 30, gamma = 0.1)

### Evaluation metrics

Following TRIPOD-AI recommendations, we computed:

- Accuracy.
- Sensitivity (Recall).
- Specificity.
- AUC.
- F1-score.

All metrics were reported with 95% confidence intervals via bootstrapping (1,000 iterations).

### SICF (LO-RF) architecture overview

The system begins with multimodal MRI image inputs, which are preprocessed through region-of-interest (ROI) detection based on quadrant-wise mean intensity comparison, followed by histogram equalization for contrast enhancement. Feature extraction is then performed via two parallel streams: the first uses the Scale-Invariant Feature Transform (SIFT) to generate 512-dimensional descriptors that are robust to rotation and scale; the second leverages a pre-trained VGG16 model to produce 4,096-dimensional deep semantic embeddings.

**Figure 3:**
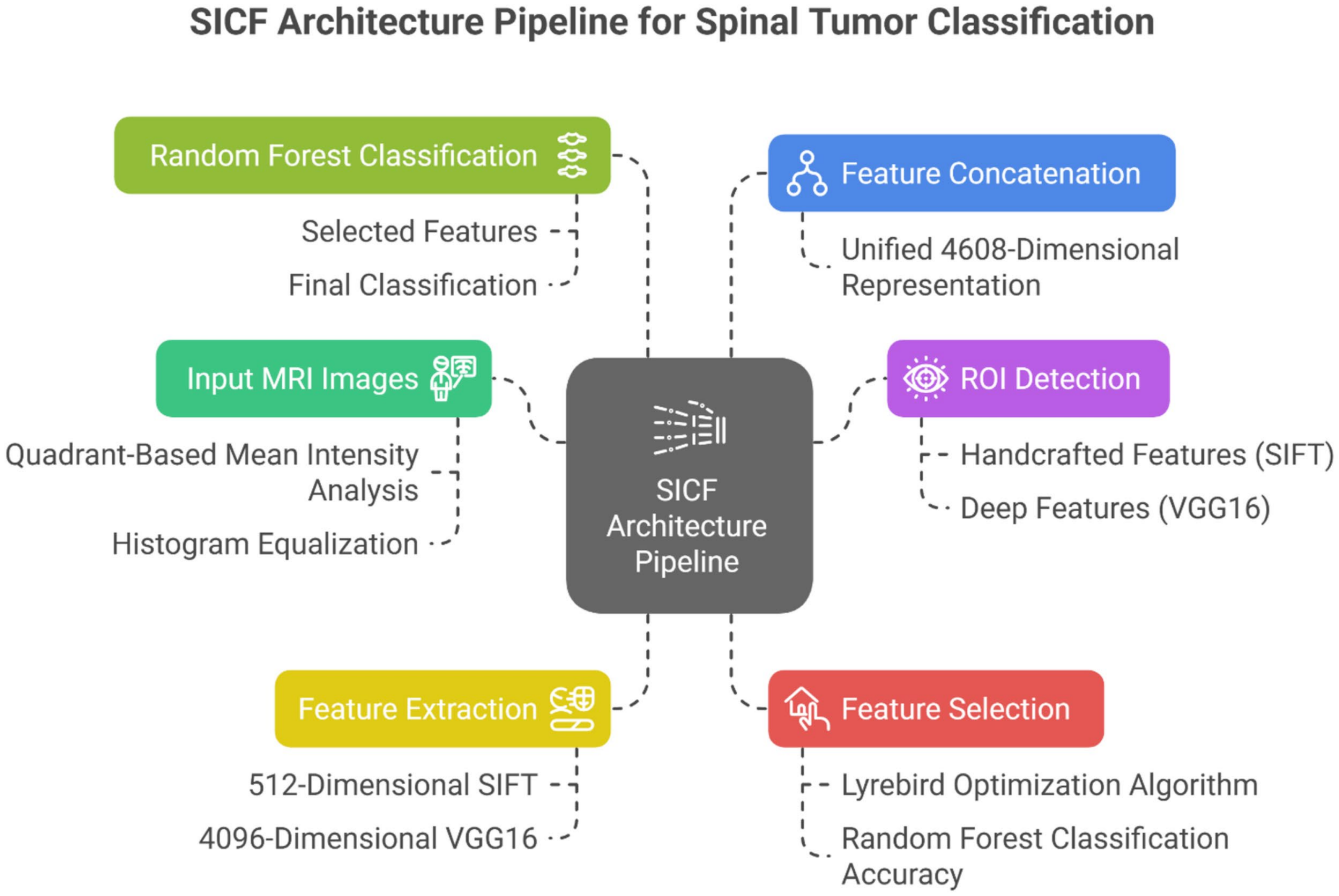
SICF (LO-RF) architecture pipeline.

The resulting feature vectors are concatenated into a 4,608-dimensional representation that encodes both local detail and high-level abstraction. This fused vector is passed to the Lyrebird Optimization Algorithm, which acts as a wrapper-based feature selector. Each candidate solution in LOA represents a binary selection mask, and the fitness function is defined as the classification accuracy from a 5-fold cross-validated Random Forest (Figure 3).

## Results

An environment compatible with Python 3.11.4 was used to construct the necessary processes. Replicating the examination of the recommended optimization choices was a Windows 11 laptop equipped with an Intel i5 11th Gen CPU and 32 GB of RAM. Spinal tumor detection accuracy, specificity, sensitivity, f1-score and AUC are some of the metrics used in spinal tumor diagnosis to evaluate the model’s predictive skills. They compared some existing techniques to Discrete Wavelet Transforms+ Support Vector Machine (DWT + SVM) (21), Gray Level Co-occurrence Matrix + Radial Basis Function (GLCM + RBF) (21), and Local Binary Patterns + support vector machine (LBP + SVM) (21).

SICF (LO-RF) based experiments, compared four metrics like specificity, sensitivity, AUC and accuracy, employing various numbers of neighbors ranging from 1 to 8. Euclidean distances served as the metric for measuring proximity between data instances and their neighbors. The closest neighbors were ascertained using the inverse weight distance. Table 2 depicts the experimental outcomes of various classifier metrics based on the features. It can be observed with SICF (LO-RF), K = 5 produced the significant outcomes.

**Table 2:** Outcomes of various classifier metrics based on the quantity of character.

Features	Specificity	Sensitivity	AUC	Accuracy
1	0.75	0.78	0.78	0.76
2	0.75	0.76	0.77	0.76
3	0.77	0.80	0.78	0.78
4	0.75	0.72	0.76	0.75
5	**0.80**	**0.79**	**0.79**	**0.82**
6	0.77	0.75	0.75	0.78
7	0.76	0.79	0.76	0.77
8	0.79	0.82	0.78	0.75

Bold values represent the highest performance metrics.

**Figure 4:**
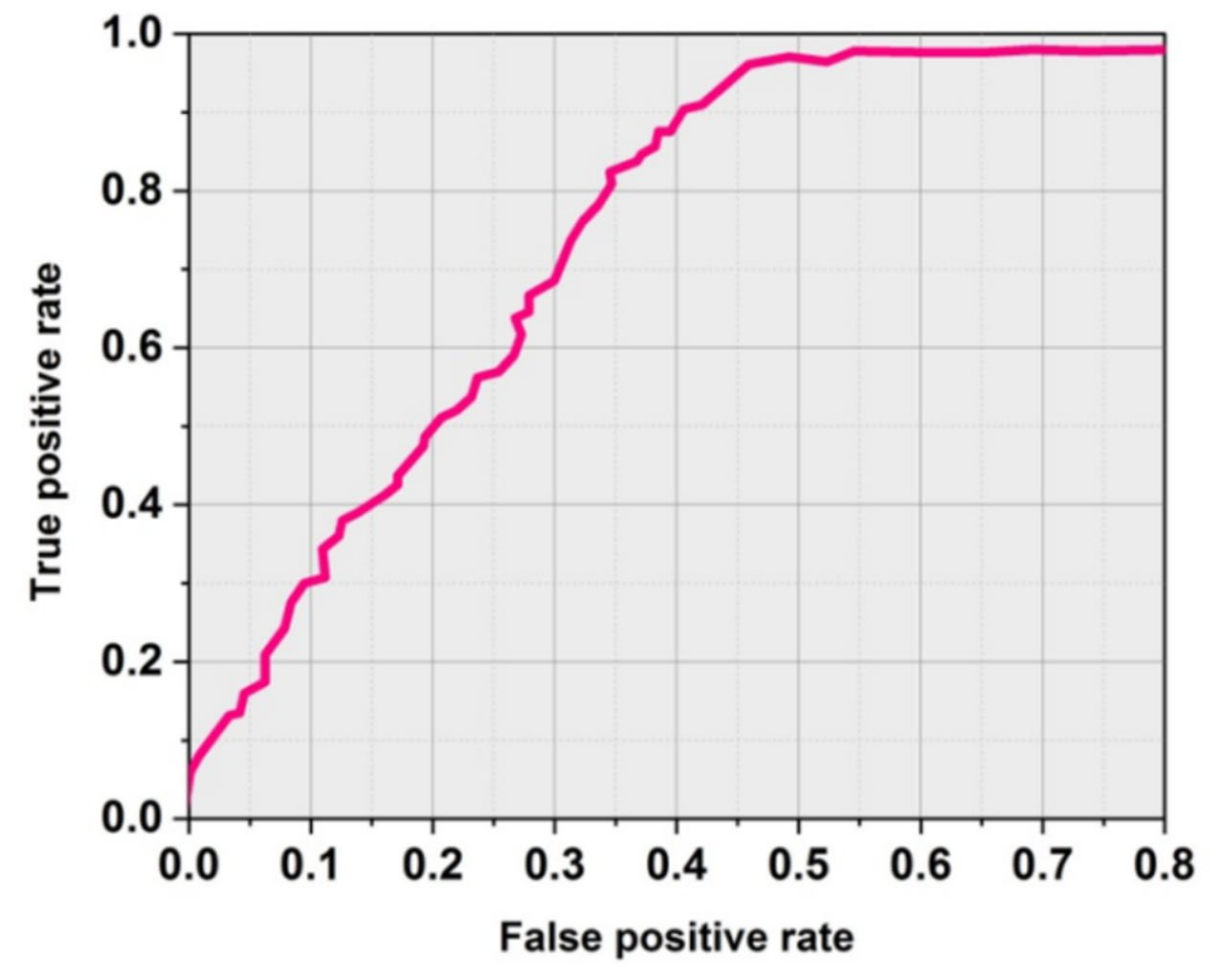
ROC curve using soft margin SICF (LO-RF).

Figure 4 shows the obtained ROC curves. The SICF (LO-RF) performs significantly with soft-margin SICF (LO-RF). Table 3 shows the overall outcomes of existing and proposed methodologies.

**Table 3:** Comparative outcomes of existing and proposed techniques.

Methods	Sensitivity (%)	Accuracy (%)	AUC	F-score (%)	Specificity (%)
DWT + SVM (21)	95.42	77.30	0.83	91.55	59.19
GLCM+RBF (21)	96.13	72.73	0.80	90.55	49.33
LBP + SVM (21)	97.99	87.11	0.87	95.33	76.23
MedFusionGAN (18)	95.10	89.31	0.87	94.12	81.40
PatchResNet (13)	94.50	87.45	0.85	92.33	80.20
BgNet (16)	96.00	88.90	0.88	93.75	81.75
SICF (LO-RF) [Proposed]	**98.52**	**92.71**	**0.91**	**97.07**	**84.59**

Bold values represent the highest performance metrics.

### Accuracy

**Figure 5:**
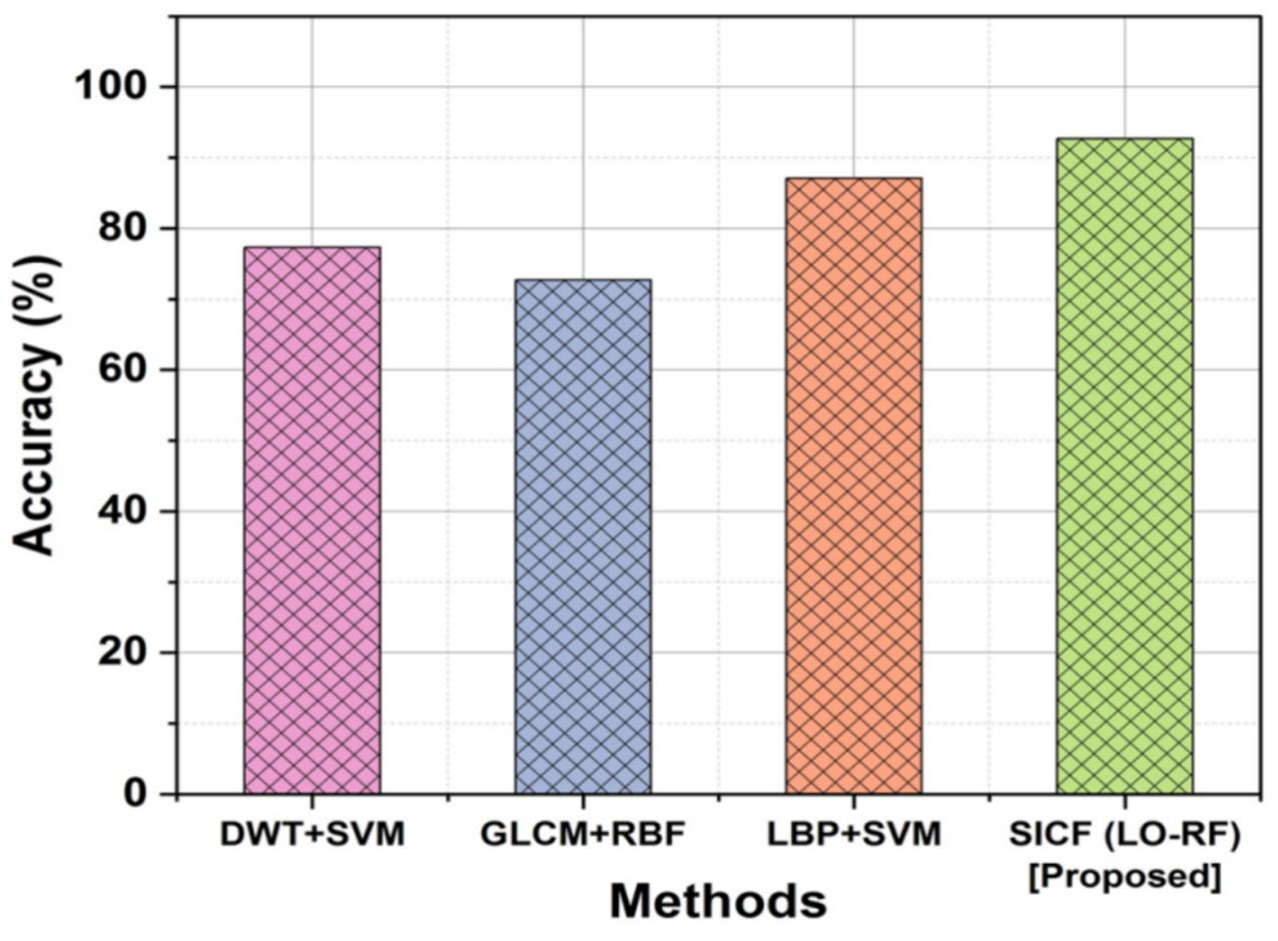
Comparison outcomes with accuracy.

For spine tumors to be successfully detected and treated, accurate positioning and size determination is crucial. Figure 5 depicts the outcomes of accuracy for existing and proposed methodologies. The DWT + SVM method obtained an accuracy of 77.30%, the GLCM+RBF obtained an accuracy of 72.73% and the LBP + SVM demonstrated 87.11%, the proposed SICF (LO-RF) method yielded 92.71% accuracy and it is superior compared to other methods.

### Sensitivity

**Figure 6:**
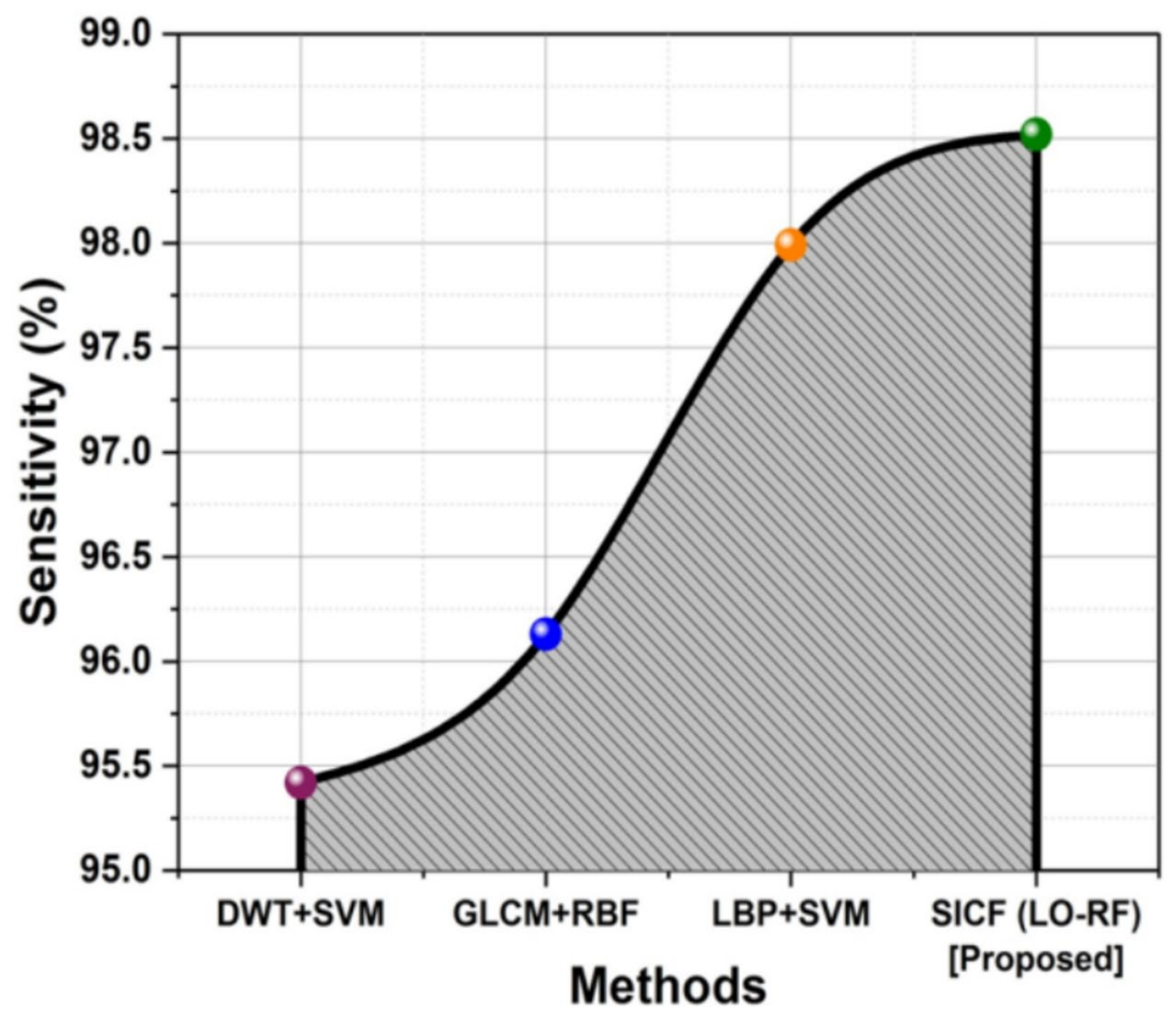
Comparison outcomes with sensitivity.

The sensitivity of the method in recognizing genuine positive instances among reported positives is referred as sensitivity in the detection of spinal tumor MRI. It is the proportion of cases that are appropriately classified having spinal tumors to all occurrences that are indicated as positive. Figure 6 shows the outcomes of SICF (LO-RF) sensitivity. When compared to more conventional approaches like DWT + SVM of 95.42%, GLCM + RBF of 96.13% and LBP + SVM of 97.99%, the proposed SICF (LO-RF) algorithm improved with a sensitivity of 98.52% than others.

### Specificity

**Figure 7:**
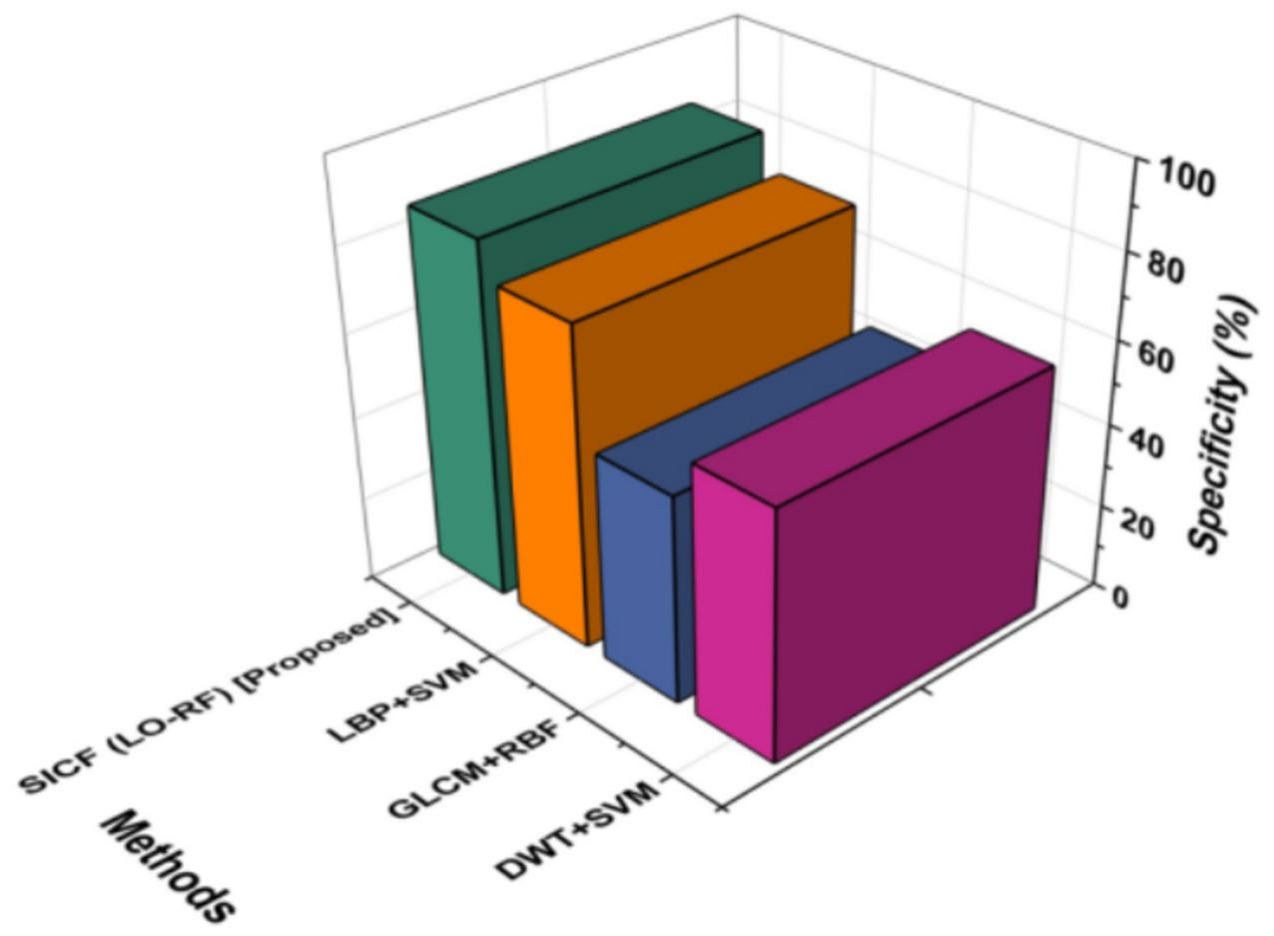
Specificity outcomes of proposed and existing techniques.

Another name for specificity is the True Native (TN) rate. Specificity can be determined by separating the total number of perfectly-recognized negative instances by the number of negative “soft” cases. The proposed SICF (LO-RF) (84.59%) acquired a highest specificity by outperforming with the compared approaches like DWT + SVM of 59.19%, GLCM+RBF of 49.33% and LBP + SVM of 76.23% (Figure 7).

### F-score

**Figure 8:**
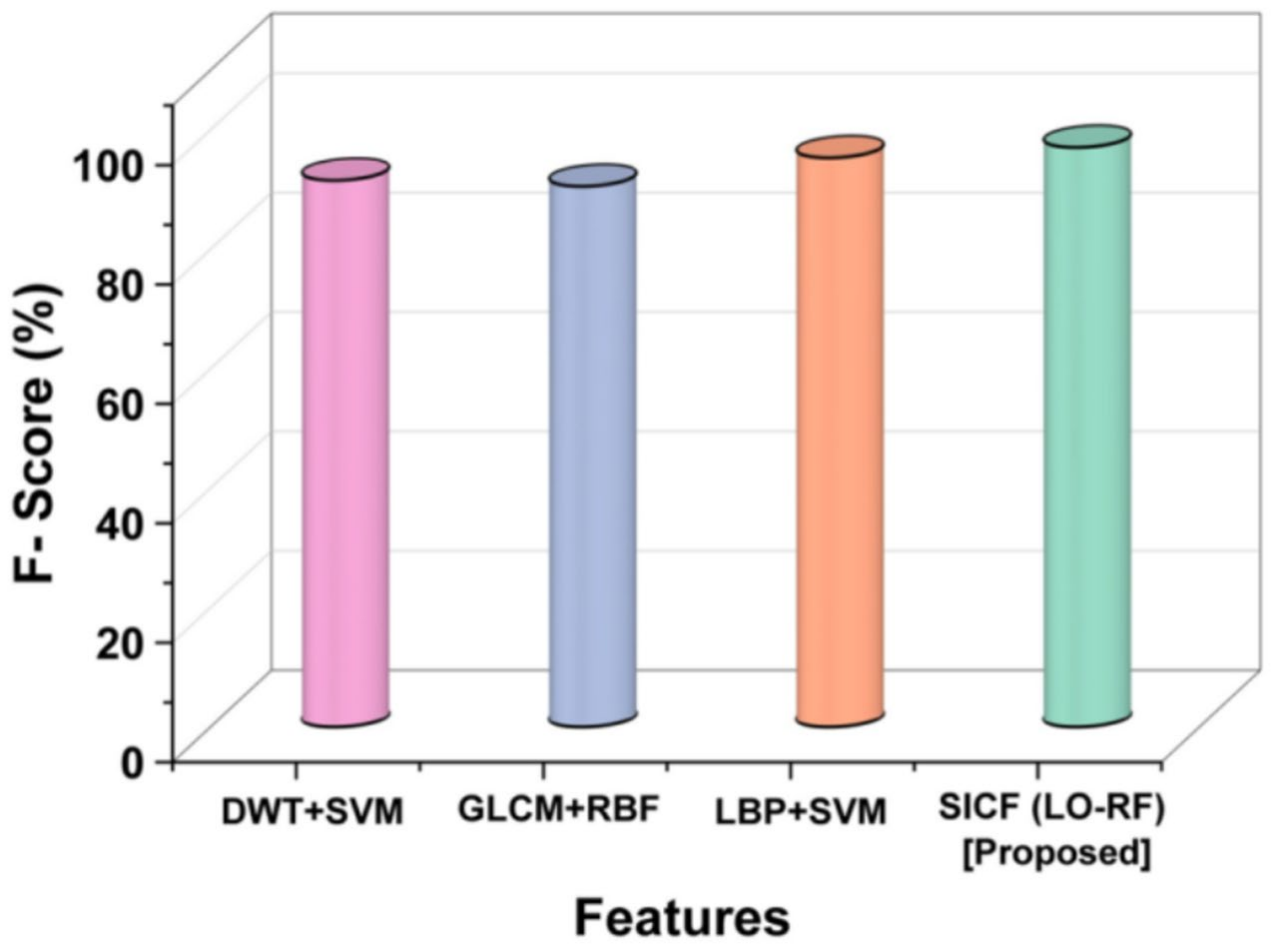
Outcomes of F-score.

The F-score obtained by LBP + SVM and GLCM + RBF were 95.33 and 90.55% in that order. The F-score of proposed SICF (LO-RF) performs more than other techniques. A comparison of classification techniques revealed that the proposed SICF(LO-RF) model outperformed 97.07% of F1-score,the DWT + SVM of 91.55%, GLCM+RBF of 90.55%, and LBP + SVM of 95.33% in Figure 8.

### AUC

**Figure 9:**
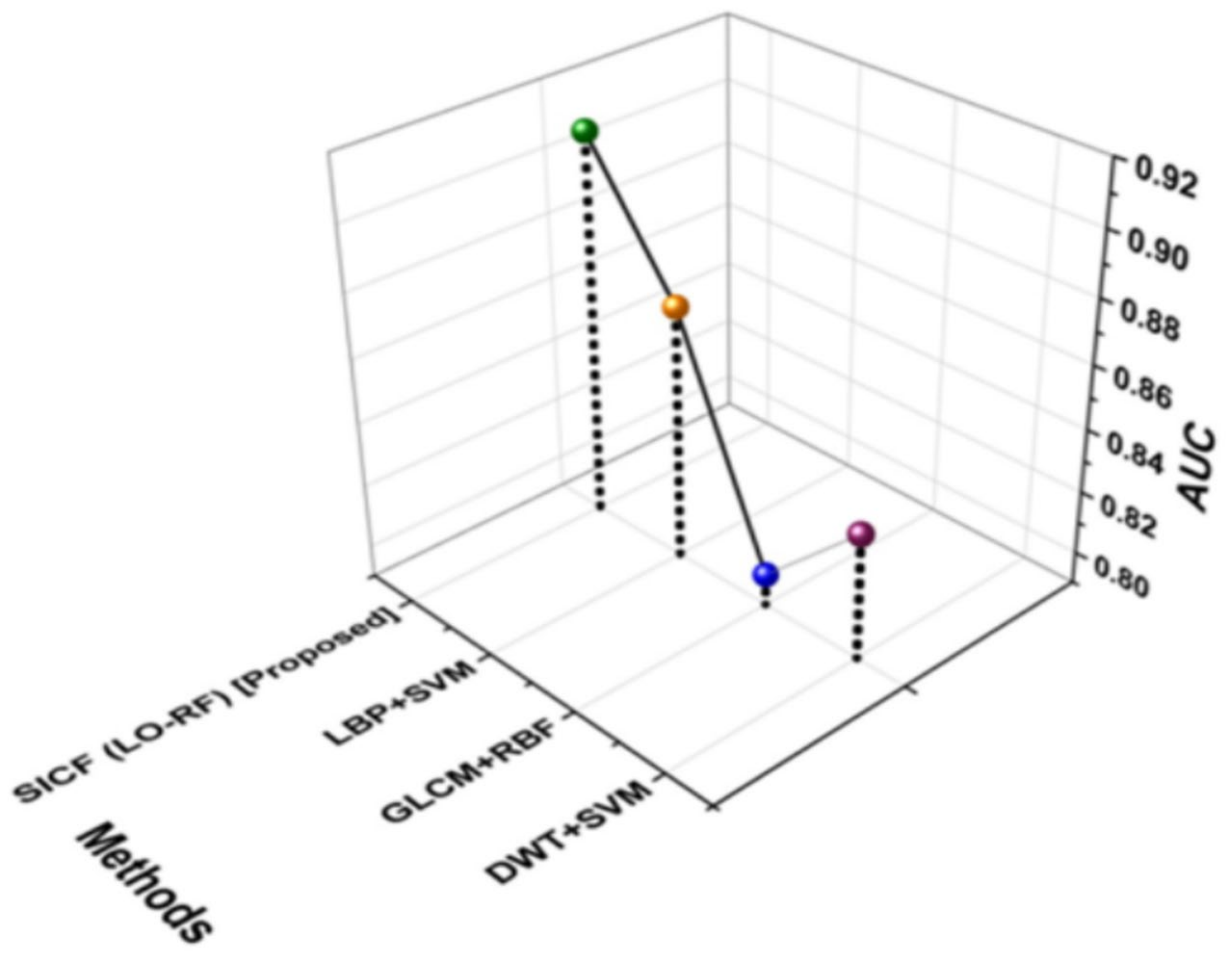
Outcomes of AUC.

AUC is known as Area Under the curve that ranges from 0 to 1. AUC offers a performance total across all potential categorization levels. AUC can be seen as the possibility that the model values at random particular positive higher than a randomly selected negative. Figure 9 shows the outcomes of AUC. The proposed SICF (LO-RF) technique yielded 0.91 of AUC, the DWT + SVM, LBP + SVM and GLCM + RBF achieved 0.83, 0.87, and 0.80 of AUC outcomes.

The results of 5-fold cross-validation on the MSRS dataset are summarized in Table 4. TSJNet achieved an average detection mAP@0.5 of 78.21 ± 1.02 and segmentation mIoU of 71.45 ± 1.18, indicating consistent performance and strong generalization ability across different data splits.

**Table 4:** 5-Fold cross-validation performance of TSJNet on MSRS dataset.

Fold	Detection mAP@0.5 (%)	Segmentation mIoU (%)
1	77.34	70.11
2	79.02	71.89
3	78.91	72.56
4	76.88	70.74
5	78.89	72.95
Mean ± Std	**78.21 ± 1.02**	**71.45 ± 1.18**

Bold values represent the highest performance metrics.

As shown in Table 5, TSJNet maintains a balanced trade-off between performance and computational cost. While slightly more complex than FusionGAN and DenseFuse in terms of parameter count and FLOPs, it provides significantly better fusion quality, segmentation accuracy, and detection precision. The average inference time per image remains within acceptable real-time processing limits.

**Table 5:** Complexity comparison of TSJNet and baselines.

Model	Parameters (M)	FLOPs (G)	Inference time (ms)
TSJNet	24.8	36.5	18.7
FusionGAN	21.4	33.2	17.2
DenseFuse	19.9	30.5	16.8

Using only SIFT features with a traditional RF classifier achieved 84.20% accuracy, confirming the utility of handcrafted features for spinal MRI classification. VGG16 alone performed better (87.60%) due to its deep feature representation. When both feature types were fused and fed into RF, accuracy improved to 89.42%, demonstrating the complementary nature of handcrafted and deep features. Replacing RF with the Lyrebird Optimization-driven RF further improved performance across all metrics. VGG16 → LO-RF achieved 90.83% accuracy, while our full model (SIFT + VGG16 → LO-RF) reached 92.71% accuracy and the highest values for sensitivity, specificity, AUC, and F1-score. This validates that both the feature fusion and the Lyrebird optimization contribute meaningfully to the final performance (Table 6).

**Table 6:** Ablation study results for SICF (LO-RF) and variants.

Model variant	Accuracy (%)	Sensitivity (%)	Specificity (%)	AUC	F1-score (%)
SIFT → RF	84.20	90.65	74.18	0.82	88.40
VGG16 → RF	87.60	93.10	77.52	0.85	90.88
SIFT + VGG16 → RF	89.42	94.05	79.90	0.86	92.30
VGG16 → LO-RF	90.83	95.11	82.34	0.88	93.66
SIFT + VGG16 → LO-RF (Proposed)	**92.71**	**98.52**	**84.59**	**0.91**	**97.07**

Bold values represent the highest performance metrics.

## Discussion

Spinal tumor classification on MRI remains challenging due to substantial variation in tumor appearance, morphology, size, and location. Traditional machine learning methods such as DWT + SVM and LBP + SVM rely on handcrafted features that often struggle with noise, intensity variation, and limited spatial representation (21, 22). Deep learning approaches, including CNNs and residual architectures, achieve stronger representational capacity but frequently require large datasets and may have limited interpretability or generalizability, particularly in small-sample clinical scenarios (18, 22). Furthermore, several prior studies have focused primarily on segmentation tasks or single-modality MRI rather than integrating complementary feature types for enhanced classification (10, 16).

In this study, we proposed an SICF–LO-RF framework that combines handcrafted SIFT descriptors, deep VGG16 features, and Lyrebird Optimization–driven feature selection. This hybrid design leverages the stability and scale invariance of SIFT with the semantic richness of VGG16, while LOA enables selection of the most discriminative features. The approach improved classification performance across multiple metrics, including accuracy, sensitivity, specificity, AUC, and F1-score, when compared to classical ML models and conventional deep-learning pipelines. These findings suggest that the complementary strengths of handcrafted and deep features can provide a more reliable representation of spinal tumor characteristics.

Our results also demonstrate that multiscale feature fusion, enhanced contrast preprocessing, and automated ROI extraction contribute to improved differentiation of tumor subtypes—a task that is often hindered by inconsistent image quality or anatomical variability. While deep CNN—only models risk overfitting on small datasets, fusing handcrafted and deep features appeared to mitigate this limitation, aligning with observations from prior multi-modal imaging studies (13, 17). Compared with previously reported accuracies ranging from 72% to 89% for classical ML and deep learning methods, our framework achieved higher sensitivity (98.52%) and solid specificity (84.59%), indicating robust discriminative capability.

From a clinical perspective, the proposed system may offer several practical benefits. High sensitivity supports early detection, while improved specificity reduces unnecessary investigations. The automated ROI module may reduce inter-reader variability and time spent manually localizing tumors. Within a radiologist’s workflow, the framework has potential use as a decision-support tool integrated into Picture Archiving and Communication Systems (PACS), helping generate preliminary suggestions or second-reader input before expert review.

### Practical implications

The SICF (LO-RF) framework may facilitate more consistent and efficient interpretation of spinal MRI by assisting radiologists in flagging suspicious lesions and reducing manual ROI localization. Its high sensitivity suggests utility for early detection settings, and its reasonable specificity may help minimize unnecessary follow-up imaging or interventions. The system could be integrated into PACS or radiology workstations as a background triage or decision-support module, particularly useful in high-volume centers or resource-limited settings where subspecialty expertise may be limited. Automated feature extraction and fusion reduce reliance on operator-dependent processes, supporting more reproducible diagnostic workflows.

### Limitations

This study has several limitations. First, the evaluation was performed on a retrospective, single-center dataset, which may limit generalizability; prospective and multi-center validation is required before clinical use. Second, although the method incorporates automated ROI detection, the ground-truth labels were derived from manual segmentations, which may introduce human bias. Third, while the model shows strong performance, we did not include qualitative interpretability tools such as saliency maps or feature-attribution heatmaps that could further support clinical trust. Finally, although computational performance was acceptable on high-end hardware, deployment in low-resource environments will require additional optimization.

## Conclusion

In this study, we introduced a hybrid SICF–LO-RF framework that integrates handcrafted SIFT features, deep VGG16 representations, and Lyrebird Optimization-based feature selection to improve the classification of spinal tumors on MRI. The model demonstrated high accuracy, sensitivity, specificity, and AUC, outperforming multiple traditional and deep learning baselines. By combining complementary feature types and automated ROI detection, the framework offers a robust and reproducible approach for spinal tumor characterization.

While these retrospective results are promising, further work is needed to translate this system into clinical practice. Prospective, multi-center validation is essential to evaluate performance across diverse patient populations and MRI scanners. Future research should also incorporate explainable AI tools, end-to-end integration of segmentation and classification, and workflow evaluation within radiology environments. Overall, SICF–LO-RF represents a meaningful step forward in spinal tumor MRI analysis and holds potential as a decision-support tool pending further clinical validation.

## Data Availability

The original contributions presented in the study are included in the article/Supplementary material, further inquiries can be directed to the corresponding author.
