# Supplementary figures and images for: An intelligent MRI data fusion framework for optimized diagnosis of spinal tumors
Source: Front Med (Lausanne). 2025 Dec 15;12:1606570. doi: 10.3389/fmed.2025.1606570 (PMC12745385; doi:10.3389/fmed.2025.1606570)

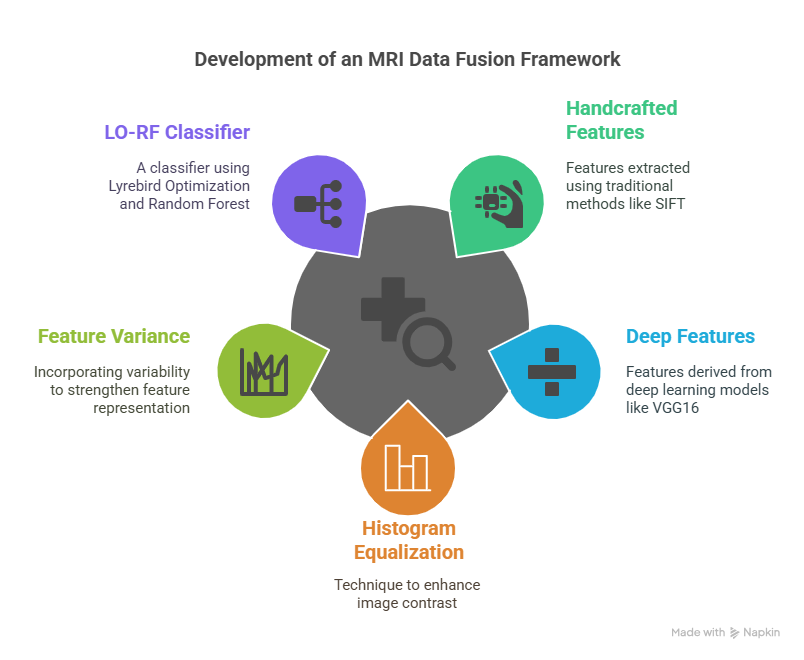

Supplement: Supplementary file 1 [file Image_1.PNG]
